# Establishment of an Artificial Tick Feeding System to Study *Theileria lestoquardi* Infection

**DOI:** 10.1371/journal.pone.0169053

**Published:** 2016-12-30

**Authors:** Shahin Tajeri, Gholamreza Razmi, Alireza Haghparast

**Affiliations:** 1 Department of Pathobiology, Faculty of Veterinary Medicine, Ferdowsi University of Mashhad, Mashhad, Iran; 2 Veterinary Biotechnology Research Group, Institute of Biotechnology, Ferdowsi University of Mashhad, Mashhad, Iran; Institut national de la santé et de la recherche médicale—Institut Cochin, FRANCE

## Abstract

The establishment of good experimental models for *Theileria* sp. infection is important for theileriosis research. Routinely, infection of ticks is accomplished by feeding on parasite-infected animals (sheep, cows and horses), which raises practical and ethical problems, driving the search for alternative methods of tick infection. Artificial tick feeding systems are based mainly on rearing ticks on host-derived or hand-made artificial membranes. We developed a modified feeding assay for infecting nymphal stages of *Hyalomma anatolicum* ticks with *Theileria lestoquardi*, a highly pathogenic parasite of sheep. We compared two different membranes: an artificial silicone membrane and a natural alternative using mouse skin. We observed high attachment rates with mouse skin, whereas *in vitro* feeding of *H*. *anatolicum* nymphs on silicone membranes was unsuccessful. We could infect *H*. *anatolicum* nymphs with *T*. *lestoquardi* and the emerging adult ticks transmitted infective parasites to sheep. In contrast, similar infections with *Rhipicephalus bursa*, a representative tick with short mouth-parts that was proposed as a vector for *T*. *lestoquardi*, appeared not to be a competent vector tick species. This is the first report of an experimentally controlled infection of *H*. *anatolicum* with *T*. *lestoquardi* and opens avenues to explore tick-parasite dynamics in detail.

## Introduction

*Theileria* are intracellular protozoan parasites which predominantly infect ruminants and are transmitted by several species of *Ixodid* tick vectors. The infection cycle starts with inoculation of 0.75–1.5 μm sporozoites by vector ticks feeding on a susceptible host. Sporozoites immediately (within 5 minutes) invade host leukocytes and develop into a syncytium structure called a macroschizont. Macroschizont-infected leukocytes become immortalized, proliferate continuously and disseminate to distant organs causing severe pathology and death of the host [1]. Further progression through the parasite life cycle involves arrest of leukocyte proliferation, leukocyte rupture and the release of merozoites. Merozoites invade red blood cells in which piroplasms are formed. Ticks take up parasitized red blood cells during feeding on an infected host. Inside the tick gut, the ingested gamonts are released from lysed red blood cells and develop into male and female gametes, which fuse to form zygotes. The zygotes invade tick gut epithelial cells and develop into motile kinetes, at a time corresponding to larval-to-nymphal or nymphal-to-adult molt of the tick. The kinete enters the tick haemolymph and migrates toward the salivary glands. The life cycle is completed by the formation of sporozoites inside the acinar cells of the tick salivary gland [2].

Infected ticks have been used in different ways in *Theileria* research; for example, Ground Up Tick Supernatants (GUTS) were used to establish lymphoid cell lines harboring the macroschizont stage of ‘transforming’ *Theileria* species [3]. GUTS were also used to infect experimental animals [4] and to maintain live parasites in the laboratory for limited periods of time. To achieve a desired number of *Theileria*-infected nymphs or adults, one should feed larvae or nymphs directly on *Theileria-*infected host, but maintenance of experimental animals is cost-prohibitive and raises ethical concerns. Furthermore, frequent experimental challenges of the hosts with ticks can induce anti-tick immunity, reducing susceptibility for future infections [5]. Hence, it is important to develop reproducible and sustainable methods for tick infection with reduced host animal use.

Several methods have been tested to infect *Rhipicephalus appendiculatus* tick with *T*. *parva* parasites, but with only partial success [6–10]. The artificial *in vitro* feeding of ticks is relatively reproducible giving a higher yield of infected ticks [11]. Currently, there are three main methods of *in vitro* tick feeding: capillary tube feeding [12, 13], artificial feeding using animal skin membranes [10, 14] or artificial feeding with cellulose paper reinforced with silicone glue [15, 16]. Capillary feeding of immature ticks is unpractical for establishing *Theileria* infection of ticks, since these stages are delicate and cannot be easily handled. Furthermore, this method has a low yield of fed ticks. Thus, membrane feeding is a more suitable method for *in vitro* tick infection with *Theileria* parasites. Several pathogens including *T*. *mutans* [17], *T*. *parva* [18, 19], *Babesia divergens* [14], *Ehrlichia ruminantium* [17], *Borrelia burgdorferi* [20] and *Bartonella henselae* [21] have been transmitted to ticks via membrane feeding. See Bonnet and Liu for an extensive review of models of *in vitro* tick infection and associated pathogens [22].

*T*. *annulata* and *T*. *parva* cause tropical theileriosis and East Coast Fever (ECF) respectively, and are among the most notorious *Theileria* species in cattle infections. The *T*. *lestoquardi* species is responsible for ovine malignant theileriosis, a fatal disease affecting sheep and goats. Large livestock populations are at risk in endemic areas, where the three diseases are considered economically important. We selected *T*. *lestoquardi* as a model for our membrane feeding studies as it has similar transmission dynamics as cattle-derived *Theileria* parasites but lower costs of purchasing and keeping host animals (i.e. sheep). Although *T*. *lestoquardi* is an understudied and neglected species, we propose that our findings can be generalized to other *Theileria* sp.

We developed a modified assay initially designed by Kröber and Guerin [15]. We reported artificial feeding of adult ticks of two different tick species, *Hyalomma anatolicum* and *H*. *dromedarii* [23], which we extend here to include *in vitro* feeding of *H*. *anatolicum* and *Rhipicephalus bursa* tick nymphs. We focused on the nymphs because these instars should take up the piroplasm stage of *Theileria* and transmit the parasite to the next tick host (trans-stadial transmission). We designed smaller feeding units and replaced the silicone membrane with mouse skin and tested the ability of artificially-infected *H*. *anatolicum* ticks to infect naïve sheep.

Ovine malignant theileriosis occurs in geographic areas where its principal vector, *H*. *anatolicum*, is not found, implying that other ticks can act as vectors [24, 25]. We tested the vector competence of the two-host tick *R*. *bursa* for *T*. *lestoquardi*. One reason for choosing *Rhipicephalus sp*. is that they are also vectors of other *Theileria* species (e.g. *R*. *appendiculatus* transmits *T*. *parva*). Furthermore, *T*. *ovis* [26–28] and *T*. *equi* [29] DNA was found in the salivary glands of *R*. *bursa* in certain epidemiological surveys, and *T*. *lestoquardi* infection was detected in the closely related species *R*. *sanguineus* [30] and *R*. *turanicus* [31]. Moreover, *Rhipicephalus* sp. are able to transmit *T*. *lestoquardi* experimentally to sheep [32] and *R*. *bursa* was proposed as a vector for *T*. *hirci (lestoquardi)* [33].

Our study highlights the potential of carefully-designed artificial feeding systems to study *Theileria* sp. infection *in vitro* and to explore tick vector compatibilities.

## Materials and Methods

### Ethics statement and experimental animals

All the clinical/laboratory experiments with the mice, rabbits and sheep were performed in accordance with Ferdowsi University of Mashhad Animal Care Committee who approved the ethical principles (No. 29500.3). The Committee strictly follows the Specific National Ethical Guidelines for Biomedical Research issued by the Research and Technology Deputy of Ministry of Health and Medicinal Education of Iran. Mice were provided from veterinary clinic of Ferdowsi University of Mashhad where they were bred. Rabbits were obtained from Mashhad University of Medical Sciences (MUMS). The sheep were bought from Abbasabad sheep breeding station, located in the north-east of Iran. None of the experimental animals died during the project. Mice and rabbits were kept in proper cages and were fed with commercial pelleted food formulated for each species. The sheep were kept in Veterinary school’s barn and were fed with alfalfa hay and concentrates. All animals were given *ad libitum* access to food and water.

### Production of nymphs of *H*. *anatolicum* and *R*. *bursa*

New Zealand white rabbits were used for propagating colonies of *H*. *anatolicum* and *R*. *bursa* [23]. To obtain nymphs approximately 1000 larvae (either *H*. *anatolicum* or *R*. *bursa*) were reared inside cotton bags fixed with zinc oxide paste to the base of the rabbit ear. The engorged larvae remained fixed to the ears and molted on-host (two-host life cycle). The newly emerged nymphs were forcibly removed from rabbit skin 7–8 days post-infestation (unfed nymphs), or 1–2 days later to have semi-fed (prefed) nymphs. Each batch of 75 collected nymphs was maintained in a glass tube in an incubator (28°C, 85% relative humidity and complete darkness). Prefed nymphs were kept in an incubator for less than two days before artificial feeding.

### Blood preparation for *in vitro* feeding of ticks

*T*. *lestoquardi*-infected and non-infected sheep blood was taken from the jugular vein in pre-heparinated (10 IU/ml, Rotexmedica, Germany) 10–20 ml syringes, and used either fresh or stored in a refrigerator for less than 48 h. The *T*. *lestoquardi*-infected donor sheep was a long-term carrier with a constant low parasitaemia of <2% throughout the study. Sheep bleeding was performed every other day; the daily volume not exceeding 20 ml. Glucose, ATP and antibiotics were not added to the blood.

### Artificial tick feeding

Two different membranes, silicone and mouse skin, were used in a tick feeding system previously described [15] with some modifications: In comparison to the feeding units designed by Kröber and Guerin [15] which we term big feeding units (BFUs), we made a smaller version with glass tubes that we call the small feeding units (SFUs). The comparative characteristics of the two feeding units are summarized in S1 Table. Such miniaturized feeding units provide a confined microenvironment imitating natural events at the host skin surface. The probability of skin infection is less than BFUs, as the area of the skin membrane is almost halved (see S1 Table). SFUs fit readily into 6-well tissue culture plates. The two outer plexiglass rings were designed smaller, accordingly. To produce mouse fur or sheep wool extract, the chopped hairs were soaked in a beaker containing 10 ml of methanol (Merck, Germany), stirred for 10 min and incubated at room temperature for 4–5 days to allow alcohol evaporation. Finally, the remaining perfume was separated from the hairs and stored in sealed glass containers (at room temperature) until use. The stopper used to close the top of the feeding chamber, in order to prevent tick escape, was a plastic ring wrapped in a cotton cloth [34]. One hour before starting tick transfer to the feeding chamber, 100 μl of sheep or mouse perfume was applied to silicone membranes with a micropipette and left to dry.

The host skin system generally resembled the one described in the previous paragraph except that the mouse skin replaced the silicone membrane. Laboratory mouse of approximately 4–5 weeks old, without any previous history of contact with parasiticides was euthanized by chloroform. After that, the hair of the mouse was partially clipped with fine scissors, leaving 3–4 millimeters of hair shaft intact. Finally, the mouse skin was attached firmly to the borders of the glass/plexiglass chamber by cyanoacrylate adhesive (Incredible Drop®, Japan tech.) not the silicone paste. A detailed pictorial guide for the aforementioned steps is available in S1 Fig, to illustrate the methodology.

Each feeding unit was fixed in a one well of a six well tissue culture plate pre-filled with 3 ml warm blood and floated on a water bath adjusted to 37°C.

### *In vitro* tick infection by *Theileria*

**Fig 1:**
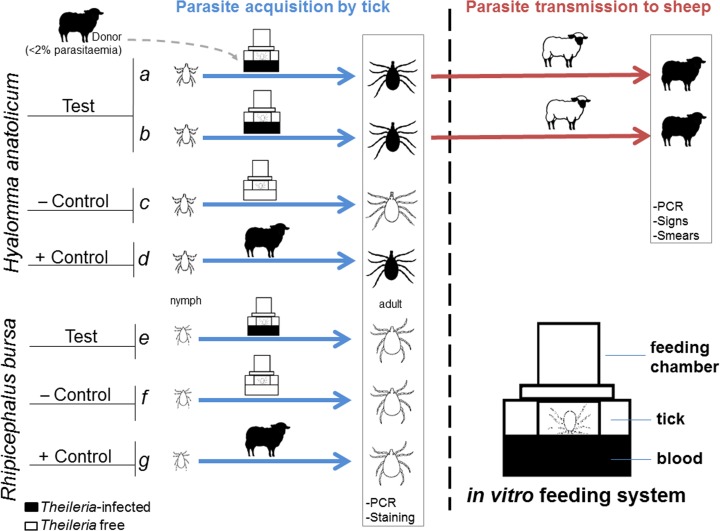
Summary of the experiments conducted in this project. Test group (experiment {*a*}): Nymphs of *H*. *anatolicum* were fed *in vitro* with infected blood obtained from the initial donor sheep. Emerged adults were tested for *T*. *lestoquardi* infection by staining the salivary glands and PCR detection of the parasite DNA. When infection was confirmed in a cohort of ticks, the rest were used to infect a healthy sheep that was monitored clinically. Test group (experiment {*b*}): This was a replicate of experiment {*a*} in which nymphs were artificially infected with blood taken from the sheep that was infected by *in vitro* fed ticks in experiment *(a)* and adults resulting from these instars were transmission fed on another sheep. Test group (experiment {*e*}): *T*. *lestoquardi* infected blood was provided for *R*. *bursa* nymphs feeding under artificial conditions. Negative control groups (experiments {*c*} and {*f*}): *H*. *anatolicum and R*. *bursa* nymphs were engorged with uninfected blood of a healthy sheep. Positive control groups (experiments {*d*} and {*g*}): Experiments were performed simultaneously. Nymphs of *H*. *anatolicum* and *R*. *bursa* fed on ears of the donor sheep. Here, *H*. *anatolicum* ticks only acted as positive controls for *T*. *lestoquardi* acquisition.

Acquisition experiments included feeding of *H*. *anatolicum* nymphs on sheep blood taken from three sources: The naturally infected donor sheep (experiment {*a*}), the sheep that was experimentally infected in experiment *(a)* transmission phase (experiment {*b*}) and *T*. *lestoquardi* non-infected healthy sheep (experiment {*c*}). *R*. *bursa* nymphs fed artificially on infected blood of donor sheep in experiment *(e)* and from *T*. *lestoquardi-*free blood obtained from a healthy sheep in experiment *(f)* (see Fig 1). Each *in vitro* feeding experiment comprised 150 nymphs divided in two SFUs: one with 75 semi-fed nymphs and one with 75 unfed nymphs.

### *In vivo* infection of ticks by *Theileria*

Two hundred fifty *H*. *anatolicum* nymphs that had molted two weeks before were applied inside a cotton bag to the left ear of the infected donor sheep. Simultaneously, 250 nymphs of *R*. *bursa* infested the right ear (experiments {*d*} and {*g*} in Fig 1, respectively). These groups were considered as positive controls for the *in vitro* feeding experiments. The cotton bags were fixed to the base of ears with zinc oxide pastes. The fully engorged replete nymphs from both ears were collected and stored in the incubator (under conditions described above) until the next molt.

### Parasite detection in ticks and animals

#### PCR

To track *T*. *lestoquardi* infection in ticks, pools comprised of 20 ticks (10 males/10 females chosen from each *in vitro* feeding unit) were processed 6-weeks after the nymphal-to-adult molt. In the case of *R*. *bursa*, a second pool was also processed 10-weeks post molt. Forty ticks fed on animals were tested. The outer surface of adult ticks was washed in detergent and their salivary glands were dissected and placed in a drop of PBS and examined under a stereomicroscope. Salivary glands from each pool were stored in 1.5 ml microtubes containing PBS and kept in -20°C until used for DNA amplification experiments.

Sheep blood was collected in tubes containing EDTA and stored in -20°C. Extraction of DNA from salivary glands or sheep blood samples was performed with a commercial kit (Molecular Biological System Transfer, MBST, Germany/Iran). The DNA was then stored at -20°C. For specific diagnosis of infection in extracted DNA, we used the PCR [35], which is applicable to both ticks and sheep blood [36] by targeting a 30 kDa merozoite gene of *T*. *lestoquardi*. The PCR reaction contained 0.5 μl of each primers (10 μM), 1 μl of template DNA, 10.5 μl water and 12.5 μl Taq 2× Master Mix (Ampliqon, Denmark). The PCR running program was 3 min at 94°C, 39 cycles of 1 min at 94°C, 1 min at 65°C and 1 min at 72°C. Finally, the samples were heated at 72°C for 5 min as the final extension step. After PCR amplification samples were electrophoresed in a 1.5% agarose gel, stained with ethidium bromide and visualized under UV light.

#### Salivary gland staining

For each cohort of ticks fed either *in vivo* or *in vitro*, fresh salivary glands were extracted from 20 ticks and stained as described [37] using azure A (Sigma chemicals, Germany). Azure staining with hydrolysis was performed as follows: the salivary glands were spread on a microscopic slide and air-dried, fixed in 96% methanol (Merck, Germany) for 5 min, then soaked for 1 min in 0.1% aqueous azure A solution and finally washed gently with water and examined under a microscope.

### Parasite transmission to host

The two transmission studies were performed at different times. The sheep were both spleen-intact, aged 6–7 months and were of Iranian Baluchi breed known to be susceptible to ovine malignant theileriosis. The sheep in experiments (*a)* and (*b)* were clinically normal and their blood/lymph node biopsy smears were free from *T*. *lestoquardi* parasites. The blood smears were all subjected to a semi-nested PCR to detect *Theileria/Babesia* sp. infection [38] and no positive reactions were detected (data not shown). The dedicated area for transmission studies was completely sprayed with 10% cypermethrin solution 2–3 weeks before commencing tick challenge. Each sheep was infested with 30 adult (including 20 females and 10 males) *H*. *anatolicum* ticks applied to one ear. The ticks were selected from batches, whose *T*. *lestoquardi* infection was previously confirmed by PCR/salivary gland staining. The challenged sheep were checked daily for rectal temperatures and general health. Peripheral blood smears and prescapular lymph node needle biopsies were taken on alternate days, Giemsa stained (Merck, Germany) and examined for the presence of piroplasms and macroschizonts, respectively.

## Results

### Membrane feeding of nymphal *H*. *anatolicum* and *R*. *bursa*

In order to optimize the tick feeding assay, we initially tested *in vitro* feeding of *H*. *anatolicum* and *R*. *bursa* nymphs on silicone membranes of variable thicknesses. Results were inconclusive as the attachment rates of nymphs were quite variable and did not exceed 5% for any given experiment (data not shown). These unsuccessful attempts raised the possibility that silicone membranes are not appropriate for *in vitro* feeding of tick nymphs for these species. We therefore decided to test the feeding of nymphs from both tick species using a modified *in vitro* feeding assay with mouse skin. The mouse skin modification resulted in marked improvement; we observed attachment rates higher than 75% in all experiments (Table 1). The feeding period of unfed nymphs was longer in comparison to prefed ticks; it took 7–8 days for unfed *H*. *anatolicum* to complete the blood meal. This period was more variable for prefed ticks, ranging from 3 to 6 days until repletion. These numbers differed slightly for *R*. *bursa*. We occasionally observed blood/serum leakage, but this did not interfere significantly with tick feeding. The data regarding the *in vitro* feeding are presented in Table 1. We found that engorged nymphs of both species molted with high efficiency and the molting rates ranged from 86–97%. These results comparing different assay conditions demonstrated that the mouse skin-based tick feeding system is a significant improvement and offers an efficient method for nymphal feeding of *H*. *anatolicum* and *R*. *bursa*.

**Table 1:** *In vitro* feeding performance of *H*. *anatolicum* and *R*. *bursa* nymphs fed on skin membranes and detection of *T*. *lestoquardi*.

Group (Experiment^a^)	Tick species studied	Tick status before the feed	No. of ticks used	infection status of blood meal	No. engorged (percent)	No. molted (percent)	No. of PCR pools^d^ (+/- result)	Salivary gland infection^f^
Test (*a*)	*H*. *anatolicum*	prefed^b^	75	infected	70 (93)	68 (97)	1(+)	+
		unfed^c^	75	infected	66 (89)	61 (93)	1(+)*	+
Test (*b*)	*H*. *anatolicum*	prefed	75	infected	63 (84)	59 (93)	1(+)	+
		unfed	75	infected	60 (80)	54 (90)	1(+)*	+
Neg. control (*c*)	*H*. *anatolicum*	prefed	75	clear	67 (90)	58 (88)	1(-)*	-
		unfed	75	clear	62 (83)	55 (90)	1(-)	-
Test (*e*)	*R*. *bursa*	prefed	75	infected	60 (80)	57 (95)	2^e^(-/-)	-
		unfed	75	infected	57 (76)	45 (89)	2^e^(-/-*)	-
Neg. control (*f*)	*R*. *bursa*	prefed	75	clear	64 (85)	55 (86)	1(-)*	-
		unfed	75	clear	59 (79)	51 (87)	1(-)	-

^a^ Experiments refer to the ones defined in Fig 1.

^b^ Molted on host and fed for 1–2 days prior to in vitro feeding

^c^ Molted in incubator and was not fed before transfer to feeding units

^d^ Each pool contained salivary glands of 20 ticks.

^e^ Refers to 2 consecutive PCR tests performed on 2 pools of ticks at 6 and 10 weeks post nymph-to-adult molt.

^f^ The presence of at least one parasite infected salivary gland was enough for considering a batch of 20 tested ticks positive (+) and if all the samples were not infected the batch was negative (-).

**Fig 2:**
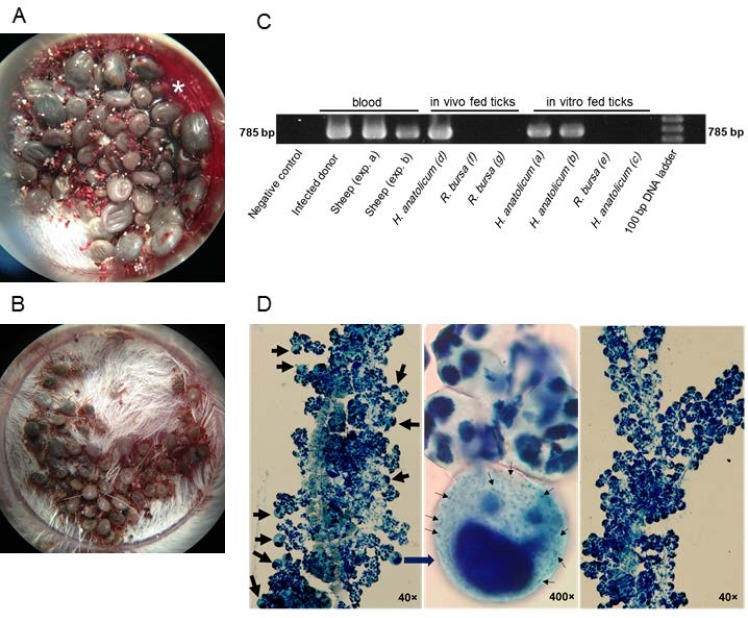
*In vitro* tick feeding and detection of *T*. *lestoquardi* in both animal and tick samples. (A) *H*. *anatolicum* nymphs clustering on the mouse skin membrane on day 6 post-tick-infestation of a small feeding unit (SFU). A small amount of blood leak (*) was observed, but was controlled by application of filter papers. (B) 3-day fed *R*. *bursa*. (C) Agarose gel electrophoresis of *T*. *lestoquardi*-specific PCR products from sheep blood samples and adult tick salivary glands. To avoid repetition, data from selected batches of ticks are presented. Positive signals were detected for the initial donor sheep and also for the two experimentally infected sheep. The alphabet letters in parenthesis in front of the samples refers to the experiments defined in Fig 1. All *H*. *anatolicum* ticks fed either naturally or artificially from infected blood, showed *Theileria* infections in their salivary glands. Parasite DNA was not amplified from any of the *R*. *bursa* groups. The *Theileria*-free sheep served as a non-infected negative control. The size of the amplicon is 785 bp. (D) Azure staining of unfed adult tick salivary glands emerged from nymphs that were engorged with *T*. *lestoquardi* infected blood meals. (Left panel) Salivary gland of a *H*. *anatolicum* fed from sheep in experiment {*b*}. *T*. *lestoquardi*-infected acini are indicated by thin black arrows. Middle panel, Magnified view of an infected acinus adjacent to uninfected acini. Numerous *Theileria* particles (small arrows) are visible inside the infected acinar cell that has a hypertrophied nucleus. Right panel, Acini of a *R*. *bursa* salivary gland were all normal and free from parasite.

* PCR results of these pools are displayed in Fig 2C.

### Acquisition of *T*. *lestoquardi* by *in vitro* fed ticks

In order to evaluate parasite transmission, we performed molecular PCR analysis and salivary gland staining following feeding. We revealed the establishment of *T*. *lestoquardi* infection in the salivary glands of *H*. *anatolicum* ticks fed on the blood of *T*. *lestoquardi* infected sheep in experiments *(a)* and (*b)*, respectively (see Fig 2. and Table 1). Having demonstrated parasite infection in the tick feeding assay for *H*. *anatolicum* (see experiment {*a*}), we repeated the test (Fig 1, experiment *b*) and confirmed the reproducibility of the *in vitro* infection by membrane feeding. The ticks engorged with uninfected blood from healthy sheep (experiments {*c*} and {*f*}) did not show any signs of salivary infections). The presence of *T*. *lestoquardi* parasites in the tick salivary glands caused hypertrophy of salivary acini that was readily observed by azure staining (Fig 2D). *R*. *bursa* adults fed as nymphs in all feeding units (experiment {*e*}) did not show any evidence of *T*. *lestoquardi* infection by either PCR analysis or azure staining (Fig 2 and Table 1). These experiments showed clearly that *H*. *anatolicum*, but not *R*. *bursa*, ticks can be infected with *T*. *lestoquardi* using an artificial *in vitro* feeding system of infected blood.

### Acquisition of *T*. *lestoquardi* by ticks fed on infected sheep

Following the success of the modified *in vitro* feeding system, we tested tick feeding on live animal hosts. After a period of 6–7 days, 191 fully-fed *H*. *anatolicum* and 175 *R*. *bursa* nymphs were harvested from the donor sheep. The resultant adults were assessed 6 weeks post nymphal-to-adult molt for the presence of *T*. *lestoquardi* infection. As expected, the DNA of *T*. *lestoquardi* was amplified from salivary glands of *H*. *anatolicum* (Fig 2C) and acinar infection was also confirmed by azure staining (data not shown). We did not perform clinical transmission studies with these adult *H*. *anatolicum* ticks, but they served as a proof of existence of active infection in the donor sheep. *R*. *bursa* ticks engorged as nymphs did not develop *T*. *lestoquardi* infection in their salivary glands (negative PCR analysis results shown in Fig 2C). Moreover, microscopic examination of stained salivary glands of 40 adult ticks did not show hypertrophy of salivary acinar cells (data not shown). *T*. *lestoquardi* was not detected in adult *R*. *bursa* ticks when they were tested again 10 weeks post-molting (data not shown). The PCR results for the whole project are presented in Fig 2C. Together, these results confirmed that *H*. *anatolicum* is susceptible to *T*. *lestoquardi* infection following tick engorgement on a parasite-harboring host, whereas the *R*. *bursa* ticks showed no evidence for *T*. *lestoquardi* infection.

### Transmission of *T*. *lestoquardi* to naïve sheep by artificially infected ticks

Finally, to test the completion of the infective cycle, we performed a series of experiments to test for parasite transmission. In the first transmission study, 6 out of 20 female ticks engorged to repletion on healthy sheep (experiment {*a*}, transmission phase). We observed a mild fever which developed at day 10 post-tick-challenge and the superficial lymph nodes became swollen. At the same time, piroplasms and macroschizonts could be detected microscopically and parasites in the blood by PCR analysis (Fig 2C). The fever subsided after one week. During the two-month follow-up tests, the sheep had a persistent piroplasm parasitaemia of less than 1% and appeared clinically normal. Ticks feeding on the second sheep (experiment {*b*}, transmission phase) experienced cold temperature on the 3rd night and all died. Therefore, 20 adult ticks (equal numbers of both sexes) from an infected batch were added to the prefixed ear bag. Five females completed their feeding after 7 days. This sheep did not show fever, but the parasites first became visible in both blood and prescapular lymph node biopsy samples on day 22 following the first round of tick challenge. *T*. *lestoquardi* infection was confirmed in blood samples 25 days post tick infestation by PCR analysis (Fig 2C). Furthermore, blood stage parasites were still detectable in Giemsa stained smears two-months later (parasitaemia <1%). Taken together, our experiments demonstrated that subclinical disease develops in clinically challenged animals that were infected by ticks bred in the artificial feeding system.

## Discussion

Here we report that earlier experiments showing *in vitro* feeding of adult *H*. *anatolicum* ticks on silicone membranes [23], can be extended and improved to examine the nymphal stage of both *H*. *anatolicum* and *R*. *bursa* in order to develop a full *in vitro* model of tick infection for *Theileria sp*. Despite numerous attempts and changing several parameters, we failed to observe *in vitro* feeding of nymphs of both *H*. *anatolicum* and *R*. *bursa* using silicone membranes. Even when using extremely thin silicone membranes, nymphal feeding was usually accompanied by blood leakage into the feeding chamber. These membranes contain relatively little silicone, and the elasticity of the membrane is severely affected. Thus, entrance of the nymphal mouth-parts into these membranes quickly resulted in longitudinal ruptures in the membrane. Nymphal feeding, if it occurred, was slow and attachment rates were low. Our results are similar to experience reported in the literature. For example, the Kröber technique was also unsuccessful in the feeding of larval and nymphal stages of other ticks, where immature instars of *R*. *appendiculatus* did not feed on silicone membranes [39]. Moreover, the feeding success of ticks varies considerably within and between research laboratories. Tick blood feeding behavior is a complex process involving many factors and silicone membrane feeding of ticks has several drawbacks. Silicone membranes do not sufficiently support feeding of ticks with short mouth parts and it is difficult to produce membranes with uniform thickness. Finally, and most importantly, silicone membrane manufacturing still requires revision and improvement. Recently a standard methodology for making uniform silicone membranes was published by Andrade *et al*. [40], which should be further validated.

The *in vitro* feeding apparatus presented here is a combination of the feeding units designed by Kröber and Guerin, and skin membranes from mice. Unlike silicone membranes, the skin does not require application of feeding stimuli. The idea of attaching skin to the bottom of feeding units enabled the use of skin membranes in artificial tick feeding. In this approach the free borders of skin are placed below blood level and therefore do not have direct contact with air, reducing membrane bacterial/fungal infections (less than 10% in our experiments). Our results show that the cyanoacrylate glue, used for fixing animal skin to the bottom of the feeding chambers, does not affect tick or *Theileria* viability, as feeding performance was not visibly affected and ticks were able to support *Theileria* development. Moreover, the cyanoacrylate glues have been used for medical and veterinary purposes, e.g. closure of skin incisions [41]. They harden more quickly in the presence of moisture (i.e. blood here) and bind tightly with the skin surface. Consequently, the feeding unit assay can be run in less than 2h. Longer times (approximately 24 hours) are required for silicone membranes, as the silicone glue has to polymerize before use.

The experimental system we developed has a number of advantages: One of our modification is the development of smaller feeding units (SFUs) compared to the big feeding units (BFUs) designed by Kröber and Guerin [16]. SFUs do not sag in the middle of the attached skin, as is common in BFUs. Sagging at the center of the host skin attached to the feeding unit causes the skin to touch the bottom of the blood-filled well, which results in skin infection and rotting. The only disadvantage of SFUs is that a smaller number of ticks can be studied.

Another important issue in the practical use of host skin-based artificial tick feeding assays is the selection of the skin membrane based on host tropism of different Ixodid tick species and even tropism of individual life cycle stage of the tick. For example, the attachment rate of adult *H*. *anatolicum* ticks increases greatly when rat skin is used instead of mouse skin (data not shown). Prior optimization of the most suitable skin for a given tick species is a prerequisite for the *in vitro* feeding unit and could improve artificial tick feeding efficiency. Another problem of skin membrane feeding systems is the requirement to sacrifice a laboratory animal, but this could be solved by proper use of slaughtered sheep/cattle skin, readily available at slaughterhouses.

The induction of subclinical *T*. *mutans* [17] and *T*. *parva* [19] infections by *in vitro* infected ticks was reported previously; In one study, sporozoite stabilates harvested from 45 artificially-infected ticks caused clinical East Coast Fever (ECF) and the authors observed equal levels of *T*. *parva* infection in the salivary glands of the host-fed and the artificially-fed ticks [42]. In our study, the mild status of theileriosis seen in experimental sheep probably stemmed from the low parasite load in artificially-infected ticks and many reasons could be responsible: (i) gradual sedimentation of red blood cells becoming inaccessible to *in vitro* feeding ticks; (ii) the low (<2%) percent of *T*. *lestoquardi*-infected RBCs in donor sheep blood. We know that for the parasite to be successfully transferred to ticks the presence of gamonts inside RBCs is crucial and this exclusively correlates with parasitaemia of the donor animal. (iii) Parasite death during storage in refrigerator; (iv) insufficient number of adult ticks used in transmission studies. Taking all these factors into consideration, our successful *in vitro T*. *lestoquardi* infection in ticks is itself noteworthy. The transmission rate of *Theileria* to ticks in this system could be improved by increasing gamont production in the host or using conditions favorable to their production, gentle blood agitation by maintenance of feeding units in shaker incubators and concentration of RBCs prior to transfer into the feeding wells. Another solution could be placing the blood above the ticks to bypass the problem of RBC sedimentation [14].

The absence of a positive PCR signal for *T*. *lestoquardi* infection in batches of *in vitro*/*in vivo* fed *R*. *bursa* could be due to a low level of parasite burden in these ticks, below the detection limits of the test. Apicomplexan parasites such as *Plasmodium* and *Theileria sp*. face population bottlenecks during passage through their invertebrate vectors. For example, tens of thousands of *Plasmodium* gametocytes can be ingested during a mosquito blood meal, but normally just 50–100 ookinetes are produced; from these, typically fewer than five survive to produce oocytes on the midgut wall [43]. In the case of *T*. *parva*, when a female *R*. *appendiculatus* nymph imbibes blood of an animal with 5% parasitaemia, it is estimated that circa 2×10^7^ piroplasms are ingested [44]. Despite intake of a large number of parasites, rarely a hundred acini are infected in an adult tick. It is clear that there is already a large attrition of parasites in the tick, or failure to develop through the intra-tick life cycle stages [45]. Keeping in mind the low level of infection that was established using *in vitro* fed ticks and the pressure of bottlenecks on parasite numbers that *T*. *lestoquardi* probably faces during development in *R*. *bursa*, one could extrapolate that insufficent parasites (gamonts) remain to establish infection of tick salivary glands. Moreover, superior immune defense mechanisms of this tick species compared to *H*. *anatolicum* could also play a role. We surmised that development of *T*. *lestoquardi* in *R*. *bursa* may take longer than in *H*. *anatolicum* and we therefore re-examined tick salivary glands 2.5 months after the molt, but still no *Theileria* infection was detected. Since similar negative results were obtained for both the *in vivo* and the *in vitro* tick feeding experiments (i.e. absence of *T*. *lestoquardi* infection), it became clear that the *in vitro* feeding system is efficient enough to identify a given tick as a potential vector for a given blood pathogen.

In conclusion, efficient *in vitro* feeding of nymphal stages of *H*. *anatolicum* and *R*. *bursa*, as well as successful production of *T*. *lestoquardi* infection in *H*. *anatolicum* using our modified artificial tick feeding system imply that it is a reliable and reproducible method to study ticks and tick-borne diseases, especially vector competence and assessment of transmission-blocking vaccine candidates. This system is extremely easy to set up and has overcome the problems associated with previous artificial feeding approaches for Ixodid ticks. It is highly likely that our system will work for other economically important hard tick species such as *R*. *appendiculatus*, provided that the most suitable host skin is incorporated into the artificial feeding system.

## Supporting Information

S1 Fig Pictorial step-by-step guide to the construction of the feeding units. (a) A 2.5 cm × 2.5 cm piece of mouse abdomen was skinned and stretched tightly on a smooth surface (e.g. glass or plastic) previously cleaned by alcohol. A few drops of sterilized phosphate buffered saline (PBS) supplemented with 100 μg/ml streptomycin and 100 units/ml penicillin was laid between the skin and the surface to prevent dehydration as well as bacterial contamination. (b) A thin layer of cyanoacrylate glue is applied to the edge of the lower inlet of the cylindrical feeding unit. (c) The feeding unit is placed over the skin and the sticky edge therefore touches the dermis. The feeding unit is pressed gently to the skin by fingers and then an object (here a fluid filled bottle) weighing 300–400 grams is placed on the feeding unit to provide a constant vertical pressure. (d) After about 10 min the object is removed and the feeding unit that is now tightly glued to the skin is turned upside down. The excessive skin around the borders of the feeding unit is trimmed with sterile tweezers and scissors. (e) The underside of the skin membrane showing vessels and subcutaneous tissues sometimes require further trimming. (f) The dermal surface of the membrane is washed in PBS (containing antibiotics, see text) at least for 20 min and several physiologic saline washes until no traces of hair and debris are seen in the washing wells. (g) Finally, the desired number of ticks are transferred to the feeding chamber and the stopper is inserted, confining the ticks near to the feeding membrane.
(PDF)

S1 Table Physical characteristics of small feeding units used in this study. The small feeding units that were designed in this study are compared with the big feeding units developed in the past.
(DOCX)
