# Supplementary figures and images for: Establishment of an Artificial Tick Feeding System to Study Theileria lestoquardi Infection
Source: PLoS One. 2016 Dec 30;11(12):e0169053. doi: 10.1371/journal.pone.0169053 (PMC5201281; doi:10.1371/journal.pone.0169053)

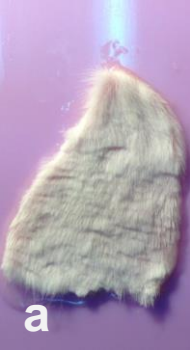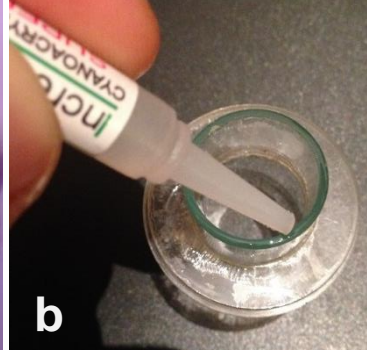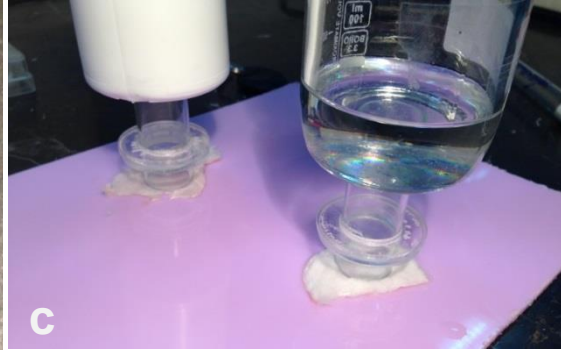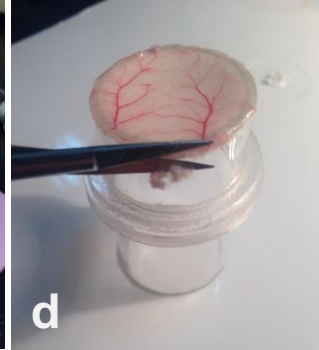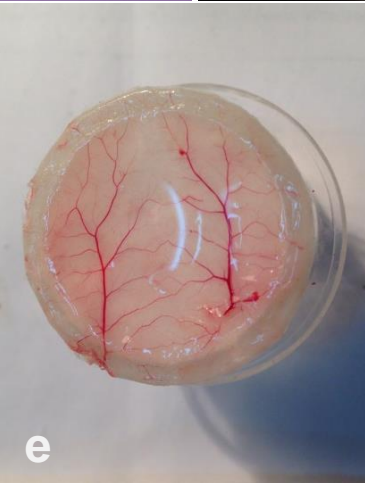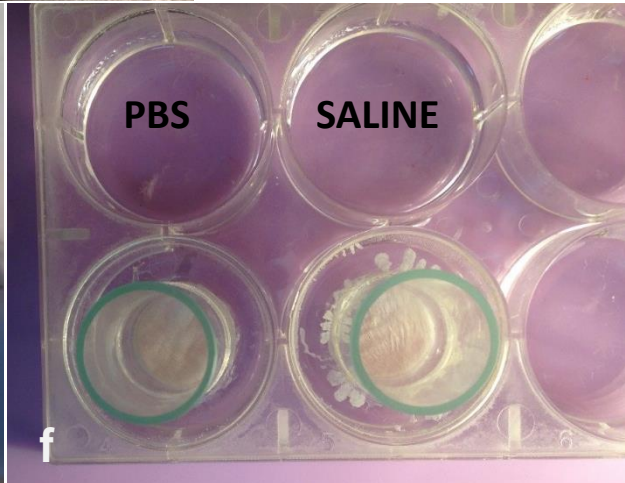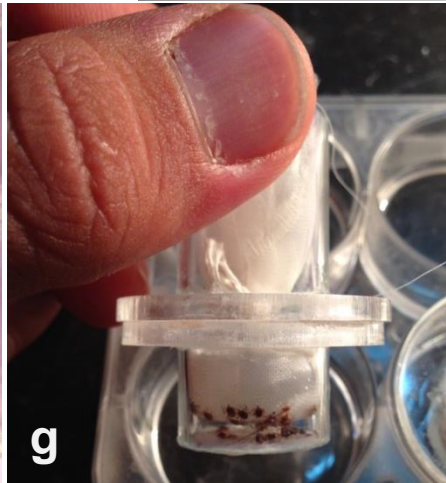

Supplement: S1 Fig — (a) A 2.5 cm × 2.5 cm piece of mouse abdomen was skinned and stretched tightly on a smooth surface (e.g. glass or plastic) previously cleaned by alcohol. A few drops of sterilized phosphate buffered saline (PBS) supplemented with 100 μg/ml streptomycin and 100 units/ml penicillin was laid between the skin and the surface to prevent dehydration as well as bacterial contamination. (b) A thin layer of cyanoacrylate glue is applied to the edge of the lower inlet of the cylindrical feeding unit. (c) The feeding unit is placed over the skin and the sticky edge therefore touches the dermis. The feeding unit is pressed gently to the skin by fingers and then an object (here a fluid filled bottle) weighing 300–400 grams is placed on the feeding unit to provide a constant vertical pressure. (d) After about 10 min the object is removed and the feeding unit that is now tightly glued to the skin is turned upside down. The excessive skin around the borders of the feeding unit is trimmed with sterile tweezers and scissors. (e) The underside of the skin membrane showing vessels and subcutaneous tissues sometimes require further trimming. (f) The dermal surface of the membrane is washed in PBS (containing antibiotics, see text) at least for 20 min and several physiologic saline washes until no traces of hair and debris are seen in the washing wells. (g) Finally, the desired number of ticks are transferred to the feeding chamber and the stopper is inserted, confining the ticks near to the feeding membrane. (PDF) [file pone.0169053.s001.pdf]
