# Supplementary material for: Establishment of an Artificial Tick Feeding System to Study Theileria lestoquardi Infection
Source: PLoS One. 2016 Dec 30;11(12):e0169053. doi: 10.1371/journal.pone.0169053 (PMC5201281; doi:10.1371/journal.pone.0169053)
Supplement: S1 Table — The small feeding units that were designed in this study are compared with the big feeding units developed in the past. (DOCX) [file pone.0169053.s002.docx]

**Table S1. Physical characteristics of small feeding units used in this study.**

|  | Dimensions (mm) of the feeding unit | | | | | Reference |
| --- | --- | --- | --- | --- | --- | --- |
|  | Height | Outer diameter | Inner diameter | Wall thickness | Membrane surface area (cm^2^) |  |
| SFU^a^ | 35 | 21 | 18 | 1.5 | 2.54 | this study |
| BFU^b^ | 45 | 30 | 26 | 2 | 5.3 | [[15](#_ENREF_15)],[[23](#_ENREF_23)] |

^a^ small feeding unit

^b^ big feeding unit
